# Efficient magnetic sorbent for extracting bisphenol A from aqueous samples

**DOI:** 10.1039/d5ra04923g

**Published:** 2025-12-05

**Authors:** Abdullah Alhendal, Jessy Shiju, Zahoor Ahmad

**Affiliations:** a Department of Chemistry, Kuwait University P. O. Box 5969 Safat 13060 Kuwait abdullah.alhendal@ku.edu.kw

## Abstract

Magnetic polymer sorbents were developed and evaluated for the extraction of bisphenol A (BPA) from aqueous solution using magnetic solid-phase extraction (MSPE) coupled with high-performance liquid chromatography with UV detection (HPLC-UV). Two sorbents were synthesized by modifying amine-terminated polydimethylsiloxane (PDMS) with 3-aminopropyltriethoxysilane (APTS): MPCNT, prepared with carboxyl-functionalized multi-walled carbon nanotubes (MWCNT-COOH), and MPTCl, synthesized without MWCNT-COOH and cross-linked with terephthaloyl chloride. Incorporation of MWCNT-COOH introduced additional π–π interactions, hydrogen bonding, and hydrophobic domains, which significantly enhanced BPA uptake. Various analytical techniques were employed to characterize the morphology, thermal properties, and particle stability of MPCNT, including X-ray photoelectron spectroscopy (XPS), zeta potential measurements, Fourier transform infrared spectrometry (FT-IR), thermogravimetric analysis (TGA), scanning electron microscopy (SEM), atomic force microscopy (AFM), and transmission electron microscopy (TEM). To optimize the extraction performance, several extraction conditions were studied, including the amount of polymer content, the pH effect, the sample volume used for both adsorption and desorption, and the salting-out effect. The reproducibility of the MPCNT-based extraction method was found to be acceptable, with a relative standard deviation (RSD) of 7.85%. The method's linearity was tested, and the limits of detection (LOD) and quantification (LOQ) were determined to be 15.15 µg L^−1^ and 50.00 µg L^−1^, respectively, with a high coefficient of determination (*r*^*2*^ = 0.9992). The relative standard deviations obtained were consistently below 10% (*n* = 5).

## Abstract 2

Schematic representation of the MPCNT sorbent and MSPE procedure.

## Graphical abstract

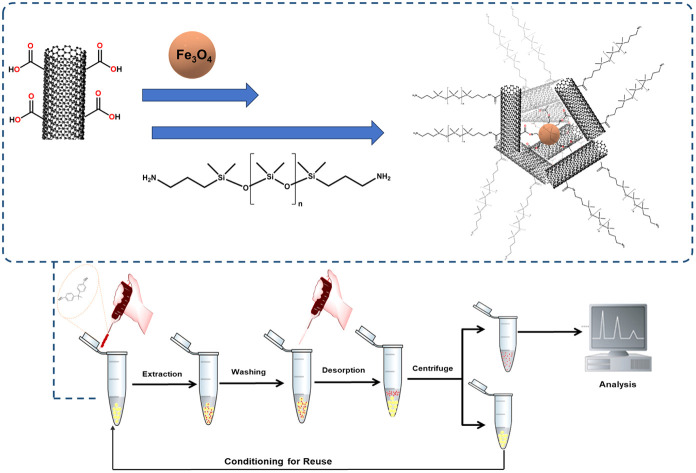

## 1. Introduction

Bisphenol A (BPA) is a chemical extensively used in the large-scale industrial production of certain plastics and resins, with its primary applications being in the manufacturing of polycarbonate plastics and epoxy resins.^1–3^ These materials are commonly found in everyday products such as water bottles, plastic containers, and baby feeding bottles, due to their excellent chemical resistance and dimensional stability.^4^ Given the widespread use of these items, it is essential to develop effective strategies for synthesizing sorbent materials capable of efficiently extracting bisphenol A from various sources.^5–7^ Several techniques have been employed for the separation and enrichment of BPA from samples, including precipitation, flocculation, filtration, and ultrafiltration. Among these, solid-phase extraction (SPE) is the most widely used and effective, offering rapid processing and good efficiency, making it suitable for use with novel solid sorbents.^8–14^ Magnetic solid-phase extraction (MSPE), however, presents distinct advantages over conventional SPE. By utilizing magnetic core adsorbents, MSPE allows for the easy collection of sorbents from the sample solution using an external magnetic field, thereby eliminating the need for labor-intensive centrifugation or filtration.^15^ This method has diverse applications, including the removal of heavy metal ions and the detection of proteins and drugs.^16–18^ Despite these benefits, developing highly efficient magnetic sorbents with magnetic properties and abundant adsorption sites remains a significant challenge for enhancing the effectiveness of the MSPE approach.

Carbon-based materials, such as pristine and functionalized carbon nanotubes (CNTs), graphene, and activated carbon, are widely used as adsorbents.^19^ Although CNTs offer unique tubular structures and strong adsorption capabilities, their effectiveness can be limited by their aspect ratio and surface area. Despite these limitations, the demand for CNT-based adsorption studies continues to grow due to their promising mechanical and chemical properties.^20–24^ CNT-reinforced composites exhibit significant improvements in mechanical stability, wear and corrosion resistance, electrical conductivity, and thermal stability, making them valuable for various applications. Magnetic multi-walled carbon nanotube (MWCNT) hybrids, which incorporate iron oxide nanoparticles, combine the distinctive properties of MWCNTs and magnetic nanoparticles (MNPs). The selection of multi-walled carbon nanotubes (MWCNTs) over single-walled carbon nanotubes (SWCNTs) was primarily driven by their higher availability, lower production cost, and greater structural stability under experimental conditions. Additionally, MWCNTs offer a larger surface area due to the presence of multiple walls, which facilitates enhanced functionalization and adsorption capacity,^25^ making them more suitable for the development of robust sorbent materials for the extraction of bisphenol A (BPA). These hybrids are highly effective adsorbents, owing to their robust mechanical properties and strong binding affinity.^26–29^ While CNTs have demonstrated good sorption capacity for a range of inorganic and organic compounds, functionalization-whether non-covalent or covalent-further enhances their sensitivity and selectivity. However, this process may also cause distortion of the graphitic structure and alter the material's physical properties significantly.^30–34^ The careful selection of CNTs based on factors like functionalization degree and aspect ratio is essential for creating efficient sorbent hybrids. Well-characterized, high-purity CNTs are crucial for preparing stable sorbents.^35^ Various functionalization techniques, including polymer wrapping, metal oxide coating, and silane modification, have been explored to improve CNT performance in adsorption studies. Polymer wrapping, for instance, introduces additional active sites, enhancing the sorption of targeted analytes, including organic contaminants and phenolic compounds.^36^ The strategic design of CNT-polymer core–shell structures ensures that the polymers remain firmly attached, even after unbound polymers are removed.^36,37^ Overall, CNTs have shown significant potential in various fields due to their ability to effectively adsorb organic pollutants.^37^

Recent studies have demonstrated the potential of magnetic nanomaterials and functionalized carbon-based hybrids in water purification due to their high surface area, ease of separation, and tunable surface chemistry. For example, Fe_3_O_4_-based magnetic nanocomposites have been reported for the removal of phenolic pollutants,^38,39^ while graphene oxide and CNT-based hybrids have shown enhanced affinity toward bisphenols and other endocrine-disrupting compounds.^40^ More recent works also highlight the use of polymer-coated magnetic sorbents and functionalized mesoporous materials to achieve high recovery and reusability. These advancements underline the importance of combining magnetic nanoparticles with carbon nanostructures for designing efficient sorbents, providing a solid basis for the MPCNT material reported in this study.^41,42^

This study explores the extraction of bisphenol A using a newly developed magnetic sorbent, which was modified with PDMS, APTS, with and without the inclusion of carboxyl-functionalized multi-walled carbon nanotubes (MWCNT-COOH). Iron oxide particles were chosen as the magnetic component due to their excellent magnetic properties, biocompatibility, and ease of functionalization. Functionalization with 3-aminopropyltriethoxysilane (APTS) was specifically employed to introduce amino groups on the particle surface, enabling further coupling with other functional molecules. This modification not only enhances the stability and dispersibility of the iron oxide particles in aqueous systems but also facilitates covalent interactions with the polymer matrix, improving the structural integrity and performance of the resulting sorbent material. The magnetic property was incorporated to facilitate an easier pretreatment process. Magnetic MWCNTs (M-CNTs) were prepared by forming amide bonds between the –COOH functional groups on the MWCNTs and the –NH_2_ terminal groups of APTS-functionalized iron oxide particles, providing stability against mechanical stress, such as sonication or extended stirring. PDMS was selected as the polymer matrix due to its unique combination of properties, including excellent thermal stability, chemical resistance, and flexibility. These characteristics make PDMS highly suitable for functionalization with amine and other groups, enabling the preparation of hybrid materials with tailored surface chemistry. Subsequently, PDMS was grafted onto the magnetic iron particles *via* amide bonding, resulting from the reaction between the –COOH groups of M-CNT and the –NH_2_ groups of PDMS, thus forming the PDMS-grafted M-CNT (MPCNT). The formation of amide or ester bonds between the –COOH groups on the CNT surface and polymers or their derivatives is a common approach for synthesizing polymer-grafted CNTs. The synthesized MPCNT was thoroughly characterized using various techniques, including Fourier-transform infrared spectroscopy (FTIR), X-ray photoelectron spectroscopy (XPS), thermogravimetric analysis (TGA), atomic force microscopy (AFM), scanning electron microscopy (SEM), transmission electron microscopy (TEM), and zeta potential analysis to confirm the successful implementation of the designed strategy. The study also optimized five key factors influencing extraction performance, while the choice of eluent and its impact on the structural integrity of bisphenol A were considered. Regeneration experiments were conducted using particles without MWCNT (MPTCl), demonstrating that the polymer-wrapped MWCNTs were reusable for up to five extraction cycles. This research anticipates that CNT-polymer hybrids will play a leading role in developing advanced sorbents for solid-phase extraction (SPE), potentially replacing many older techniques. However, the full potential of CNT-polymer combinations has yet to be fully realized.

## 2. Experimental

### 2.1. Reagents and materials

The chemicals in this study were of analytical reagent grade and were used without any additional purification, bisphenol A (BPA, ≥ 99%), poly(dimethylsiloxane) bis(3-aminopropyl) terminated (average M_*n*_ of 2500), ethanol (≥99.5%, ACS reagent), iron(iii) chloride hexahydrate (FeCl_3_·6H_2_O, ACS reagent, 97%), iron(ii) sulfate heptahydrate (FeSO_4_·7H_2_O, ACS reagent, ≥99.0%), 3-aminopropyl)triethoxysilane (APTS, 99%), ammonium hydroxide (NH_4_OH, 25%), hydrochloric acid (HCl, 37%), sodium hydroxide (NaOH, pellets), chloroform (suitable for HPLC, ≥99.8%), triethylamine (TEA, ≥99.5%), acetic acid (CH_3_COOH, ACS reagent, ≥99.7%), sodium acetate trihydrate (CH_3_COOH·3H_2_O, ACS reagent, ≥99%), terephthaloyl chloride (≥99%, flakes) were purchased from Sigma Aldrich, and acid functionalized MWCNTs (95+ %, OD:8 nm, ID: 2–5 nm and length: 0.5–2 µm) were obtained from Nano Armour. The solvent dimethyl acetamide (DMAC, ≥99.5% (GC)), acetonitrile (ACN, ≥99.9% (GC)) were analytical grade products of Sigma Aldrich.

### 2.2. Instrumentation and chromatographic conditions

Fourier transform infrared (FTIR) spectroscopy used to get the absorption spectra of the sorbent particles in the range of 400–4000 cm^−1^ were taken from JASCO FTIR Spectrometer-6300. X-ray photoelectron spectroscopy (XPS) was used to determine the surface elemental composition of C, O, Si, Fe, and N to identify the functional groups present. The XPS spectra were recorded with a Thermo ESCALAB 250 Xi using a monochromatic radiation source. Scanning electron microscopy (SEM) was used to study the morphology of the sorbent particles. To get SEM micrographs the particles were fixed on the stub by using a double-sided tape and then coated with gold Multimode atomic force microscope with nanoscope IV controller was used for atomic force microscopy (AFM) analysis. Images were acquired with the tapping mode RTESP tips. Transmission electron microscopy (TEM) samples were prepared by dispersing the sorbents and MWCNTs in ethanol and drying the sample on the grid in a vacuum. The TEM images were obtained at 120 kV with a JEOL TEM-1200ExII STEM. Thermogravimetry (TGA) was performed on approximately 10 mg of the sample from ambient to 800 °C at a heating rate of 10 °C min^−1^ in air using TGA-50 TA automatic analyzer. ZETA potential was used to test the surface charges on the sorbent particles. Zetasizer Nano Zs with MPT Titrator was used. The pH range was fixed between 3-and 12 with an increment rate of 0.5. The chromatographic analyses were performed on waters UPLC-H class system with a C_18_ column (15 mm × 2.1 mm × 1.7 µm) that was equipped with a PDA detector. Bisphenol A was analyzed at 25 °C with acetonitrile-TFA (0.1%) (40 : 60, v/v) as the mobile phase at a constant flow rate of 0.3 mL min^−1^. The detection wavelength and sample injection volume were 230 nm and 10 µL, respectively. Chromatographic data were processed using Empower software.

### 2.3. Preparation of magnetic nanoparticle (Fe_3_O_4_)

For the synthesis of Fe_3_O_4_ nanoparticles, the conventional precipitation method was used.^43–46^ First, FeCl_3_·6H_2_O (12.2 g, 0.0451 mole) and FeSO_4_·7H_2_O (8.4 g, 0.0302 mol) were dissolved in 50 mL of water. The solution was stirred for 30 min at 90 °C, after which 10 mL of NH_4_OH (25%, v/v) was added. The temperature was then increased to 80 °C, and stirring was continued for an additional 30 min. The resulting Fe_3_O_4_ nanoparticles were separated by centrifugation and dried at 60 °C. The resulting particles were washed several times with ethanol and deionized water, and finally dried at 50 °C overnight.

### 2.4. Preparation of amine functionalized Fe_3_O_4_

In the second step, APTS-functionalized Fe_3_O_4_ was prepared by dissolving 0.7 g of the previously synthesized iron particles in a 1 : 1 ethanol–water (v/v) mixture and sonicating for 2 h to ensure uniform dispersion. Subsequently, 3-aminopropyltriethoxysilane (APTS, 2.8 mL, 2.65 g) was added under a nitrogen atmosphere, and the mixture was stirred at 40 °C for 2 h. Based on the stoichiometry, the theoretical yield of APTS-functionalized Fe_3_O_4_ was calculated to be 1.368 g, with Fe_3_O_4_ contributing approximately 51.16% and APTS contributing 48.84% of the total mass. The resulting particles were precipitated, separated by centrifugation, washed several times with ethanol and deionized water, and finally dried at 50 °C overnight.

### 2.5. Preparation of MPCNT

**Fig. 1:**
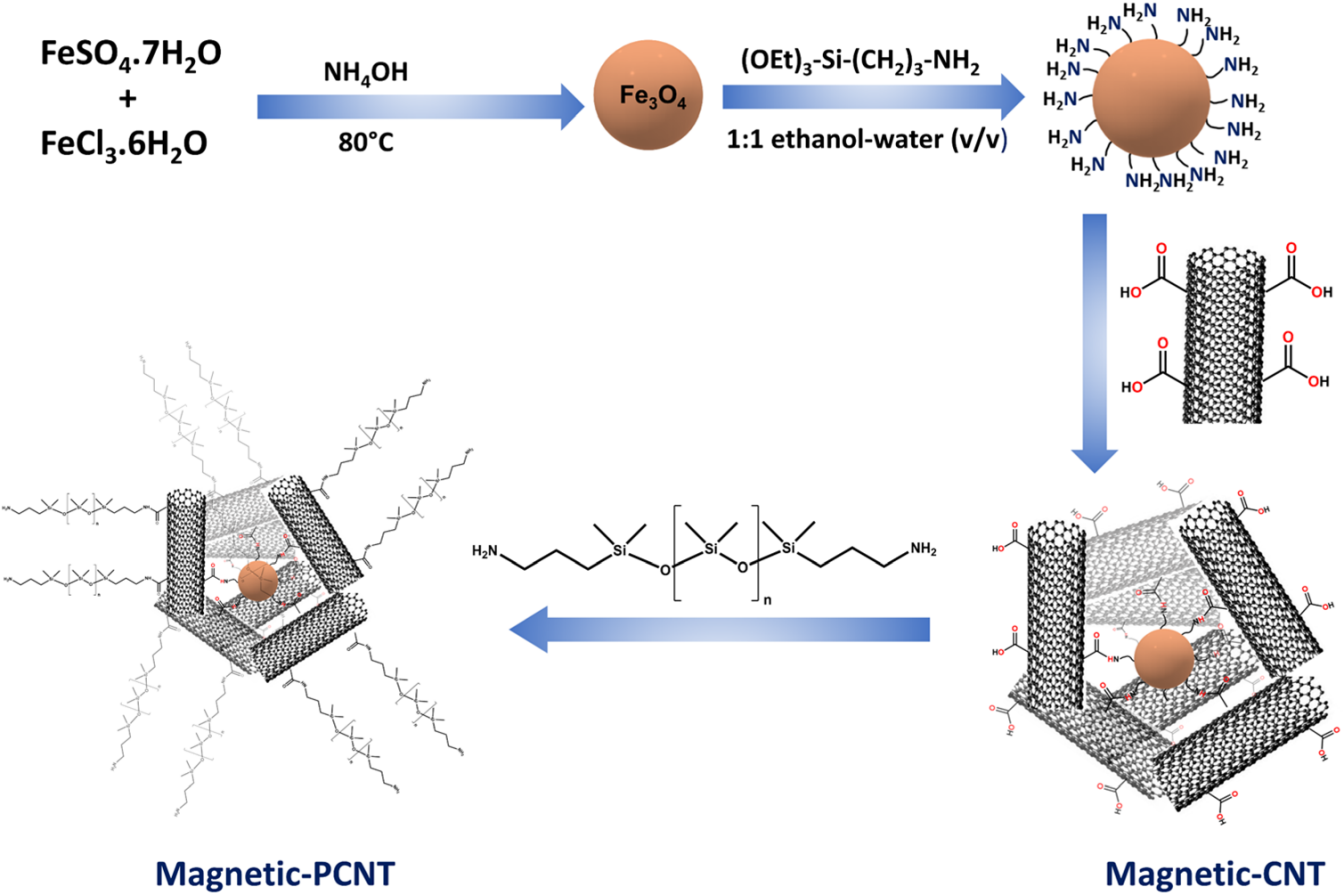
Schematic representation of the MPCNT sorbent preparation.

To synthesize MPCNT, 0.2 g of acid-functionalized MWCNT was dispersed in 60 g of DMAC and subjected to sonication for 3 h in a 100 mL flask, followed by continuous stirring for 24 h. Afterward, 0.01 g of APTS-functionalized Fe_3_O_4_ was introduced into the mixture, which was subsequently stirred at 80 °C for 3 h. The stirring was then continued overnight at room temperature to allow for the formation of magnetic CNT (MPCNT). The residual acid groups on the MWCNT were anticipated to react with the terminal NH_2_ groups of PDMS. PDMS was then added to the MPCNT dispersion, and the reaction mixture was stirred for an additional 24 h to ensure complete functionalization. The theoretical weight percentages of each component in the final product, we used the masses of MWCNT and APTS-functionalized Fe_3_O_4_, is 95.24%, and 4.76% respectively. The synthetic procedure is depicted in Fig. 1.

The synthesis method demonstrates strong potential for scalability. Key factors include: (i) use of standard, industrially accessible techniques (sonication, stirring, magnetic separation); (ii) commercially available bulk reagents (PDMS, APTS, MWCNT-COOH); (iii) covalent amide bonding between MWCNT-COOH and PDMS ensuring structural integrity and uniformity during scale-up. Batch-to-batch reproducibility (RSD 7.85% in extraction efficiency) further supports scalability without performance compromise.

### 2.6. Preparation of MPTCl

To prepare MPTCl, bis(3-aminopropyl)-terminated poly(dimethylsiloxane) (1.072 g, 50.5%) was dispersed in 60 mL of DMAC under anhydrous conditions. Next, triethylamine (0.694 g, 32.7%) was added, and the mixture was stirred for 15 min at room temperature. Following this, terephthaloyl chloride (0.3475 g, 16.4%) was introduced under a nitrogen atmosphere, and vigorous stirring continued for 3 h. APTS-functionalized Fe_3_O_4_ was then added, and the mixture was stirred for an additional 24 h. An excess amount of TEA was used to neutralize the byproduct HCl gas, ensuring a safe environment for the addition of the amine-terminated iron particles. The resulting precipitated particles were washed sequentially with chloroform (CHCl_3_), ethanol, and water, and then dried overnight at 60 °C.

### 2.7. Sample preparation and MSPE procedure

A stock solution of bisphenol A (BPA)at a concentration of 1 µg L^−1^ was prepared by dissolving 10 mg of bisphenol A in 10 mL of acetonitrile. Working solutions were then generated by diluting specific volumes of the stock solution with 0.1 M acetate buffer solution (pH 3.5), resulting in concentrations ranging from 0.05 to 1 µg L^−1^. For the extraction procedure, 40 mg of the sorbents (MPCNT and MPTCl) were suspended in 4 mL of a bisphenol A sample solution (5 mg L^−1^, pH 9) and vigorously vortexed for 30 min to enhance extraction efficiency. The bisphenol A -loaded sorbent was isolated using an external magnetic field, after which the supernatant was decanted. Desorption of bisphenol A from the sorbent was accomplished by adding 0.2 mL of a desorption solvent composed of acetic buffer and acetonitrile (30 : 70 v/v) and vortexing the mixture for an additional 30 min. Magnetic separation was applied once again to collect the desorbed aliquot, which was immediately analyzed using HPLC-UV. This procedure effectively allows for the selective adsorption of bisphenol A onto the sorbent, with magnetic separation facilitating the elimination of the sample matrix and enabling efficient desorption of the analyte for subsequent analysis.

An acetate buffer (pH 3.5) was prepared by dissolving CH_3_COOH (0.139 mol, 8.33 g) and CH_3_COONa·3H_2_O (8.8 mmol, 1.2 g) in 1 L of deionized water. This buffer was used to prepare the sample mixtures. The pH was adjusted using 0.1 M HCl. Deionized water was used throughout for solution preparation and dilution. pH measurements were performed using a digital pH meter. Acetate buffer (pH 3.5) was deliberately employed as a competitive medium to evaluate the MPCNT sorbent's performance under controlled matrix effects. This buffer introduced defined ionic strength (from CH_3_COO^−^/Na^+^) and pH conditions that mimic competitive environments where BPA coexists with interferents. While not a real-world matrix, this approach allowed systematic assessment of the sorbent's selectivity and resilience against ionic competition during method optimization.

## 3. Results and discussion

### 3.1. Characterization of sorbents

**Fig. 2:**
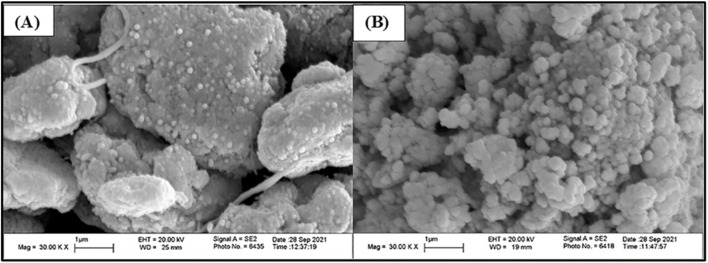
SEM image of MPCNT (A), and MPTCl (B).

The morphologies of the synthesized sorbents and the increase in MWCNT diameter resulting from polymer functionalization in MPCNT were characterized using scanning electron microscopy (SEM), and ImageJ software for determining the diameter of the prepared materials, as illustrated in Fig. 2. The MPTCl sorbent, prepared without MWCNT-COOH, displayed an almost quasi-cubical shape with a non-uniform, mixed, and irregular size distribution. This irregularity is attributed to the random orientation of the cured PDMS chains and the spatial distribution of SiO_2_ and iron oxide particles within the polymer matrix, resulting in small entities with diameters below 100 nm that tended to agglomerate. The closely packed arrangement of these particles contributed to a reduced pore density. The voids or interstitial spaces between the polymer chains and silica or iron particles are anticipated to act as active sorption sites for extraction. In the SEM images, it is evident that the MWCNT is chemically bonded to both the polymer matrix and the APTS-functionalized iron particles, yielding a more stable structure with an increased diameter and an aggregated, rough morphology for the hybrid MPCNT particles, which also exhibit larger voids throughout the matrix. Particle sizes observed in the MPCNT images range from 50 to 100 nm, where the white spherical structures correspond to both silica and broken MWCNT tips, while the dark spheres represent Fe_3_O_4_ particles. The variation in particle sizes may be attributed to differences in the aggregation of the CNTs and the extent of polymer imbibition, reflecting the integration of each polymer chain during MPCNT synthesis. The MWCNTs within the hybrid were found to be well-dispersed and enveloped by polymer chains, as shown in Fig. 2. The outer diameter of the MWCNT-COOH was approximately 8 nm in its pristine form, while SEM analysis indicates an increase to 60–90 nm due to polymer encapsulation. This microtopography revealed a highly porous globular structure for the M-CNT incorporated PDMS network hybrid, resulting from the aggregation of MWCNTs. While the MPCNT maintained the pore structure observed in MPTCl, additional channels formed between the aggregated masses. This structural feature is likely to enhance the material's surface area, thereby increasing the absorption capacity of MPCNT.^47^

**Fig. 3:**
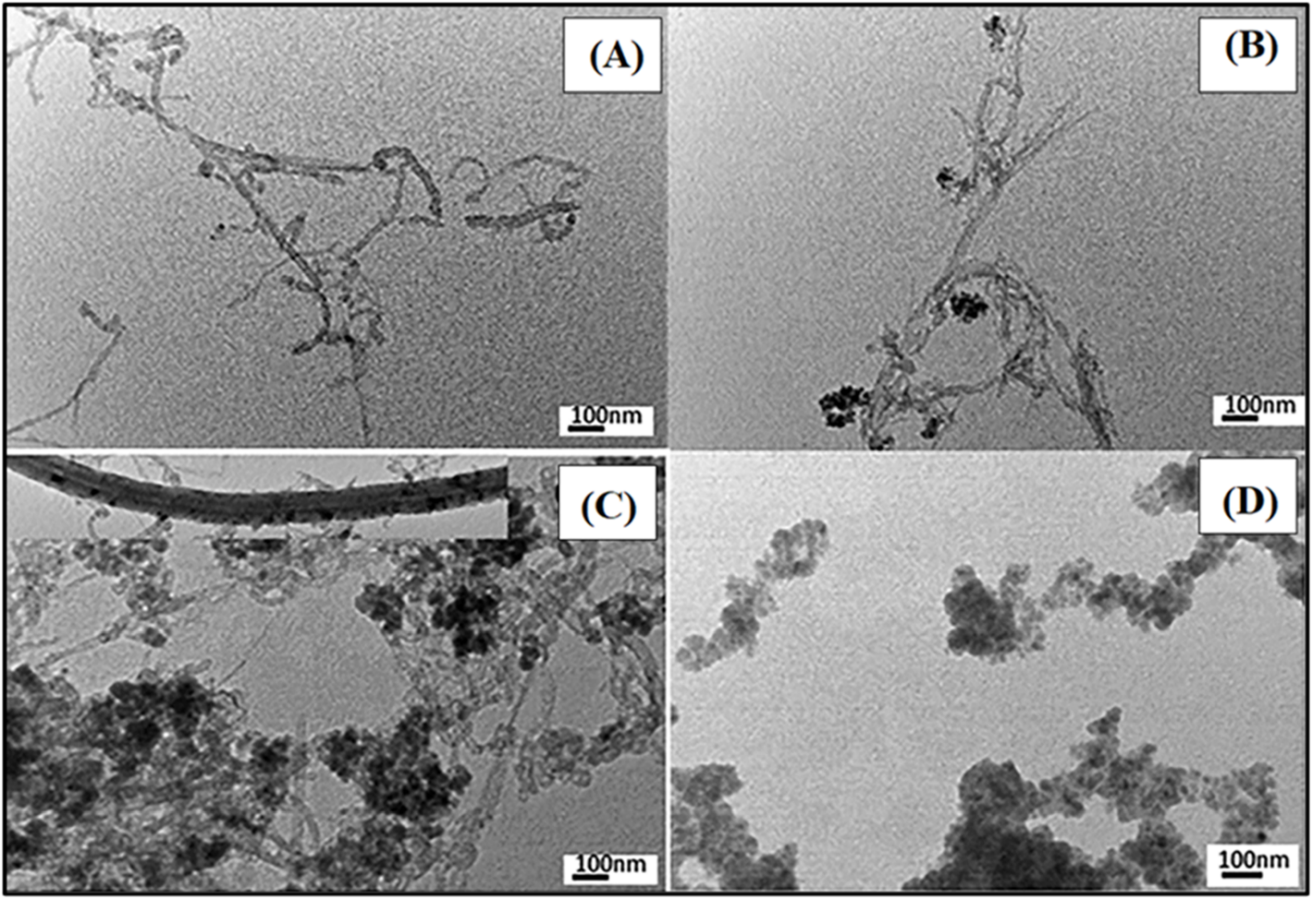
TEM images of MWCNT (A), M-CNT (B) MPCNT (C) and MPTCl (D).

The transmission electron microscopy (TEM) micrographs of MWCNT-COOH, MPCNT, and the synthesized magnetic sorbents are displayed in Fig. 3 to observe their morphologies. MWCNT-COOH had an average diameter of approximately 8 nm. Fig. 3A shows that spheroidal iron nanoparticles were chemically attached to the CNT surface in MPCNT. Following polymerization between MPCNT and PDMS, the increase in MPCNT diameter indicates that MPCNT tends to integrate (Fig. 3B), resulting in a rougher surface due to the formation of the PDMS polymer layer. A magnified view is shown in Fig. 3C to illustrate the polymer wrapping around the CNT and the iron particles within the PDMS matrix. Thus, the successful formation of the MPCNT hybrid is confirmed by the TEM analysis.^46^ Moreover, noticeable differences in morphology between the two sorbents, MPTCl and MPCNT, are revealed in Fig. 3. MPTCl appears as particles with varied size distributions, consistent with its SEM structure. The TEM micrographs of MPCNT suggest that the CNTs are isolated, well-dispersed, and form a thicker network due to the APTS-bonded iron oxide grafting and the wrapping of polymer chains on their surface.^47,48^ The outer diameter of the original CNT was around 8 nm, as provided by the supplier, while it increased to 60–100 nm in the hybrid sorbent due to surface modification. The polymer wrapping and the bound nanoparticles of silica and iron enhance the rigidity of the CNTs, which may help the sorbent restore its original pore size and rigidity after each extraction cycle.

**Fig. 4:**
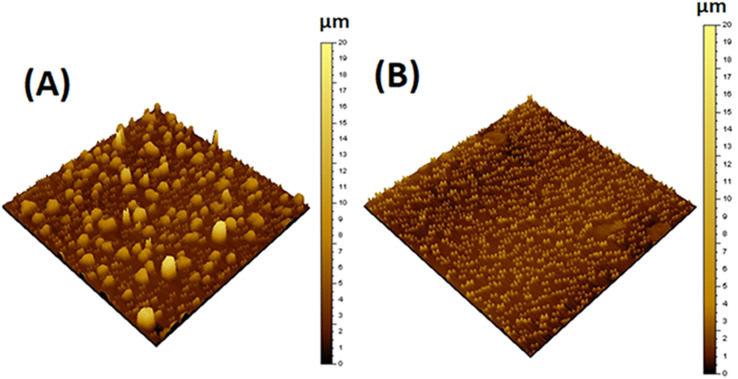
AFM images of MPCNT (A) and MPTCl (B).

AFM was used to study the surface topology of the prepared hybrids and to analyze the roughness and uniformity of CNT dispersion in the MPCNT hybrid. Fig. 4 shows the representative AFM surface topology of the hybrids. The size of the particles points out the roughness values. To check the roughness, AFM images were scanned over a surface area of 5 × 5 mm for particles, and for comparison, the vertical scales for these images were kept at 20 µm. The statistical roughness analysis of MPCNT shows that the obtained roughness or the root mean square roughness is 2.16 µm, surface skewness is 2.06 µm, the surface kurtosis is equal to 8.35 and the maximum height is 18.6 µm. The MPTCl gave comparatively lower values of roughness equal to 0.772 µm and 6.71 µm maximum height. The results showed that the surface of MPCNT nanoparticles is more rough compared to that of MPTCl which accedes to the TEM/SEM illustration.^49–51^

While SEM, TEM, and AFM analyses confirmed that MPCNT possessed a rougher surface and larger voids compared to MPTCl, quantitative surface area and porosity measurements such as BET surface area and pore size distribution were not performed in this study. The morphological evidence, together with the improved extraction performance, strongly suggests that MPCNT provides more accessible adsorption sites. However, we recognize that BET analysis would allow direct quantification of the surface area and pore characteristics, and future studies will incorporate this technique to provide complementary structural confirmation.

To quantitatively assess the dispersion of MWCNT-COOH within the PDMS matrix, TEM images of MPCNT (Fig. 3C) were analyzed using ImageJ software. The distribution of MWCNTs was evaluated by measuring inter-tube distances and counting isolated *vs.* aggregated tubes across five representative 500 × 500 nm regions. Results showed that 85 ± 4% of MWCNTs existed as isolated tubes or small bundles (<3 tubes), with an average inter-tube distance of 12.3 ± 2.1 nm. This confirms uniform dispersion and minimal agglomeration, consistent with the effective polymer wrapping observed in Fig. 3C. The absence of large aggregates (>50 nm) is critical, as agglomeration would reduce accessible surface area and hinder analyte diffusion, thereby diminishing extraction efficiency.

**Fig. 5:**
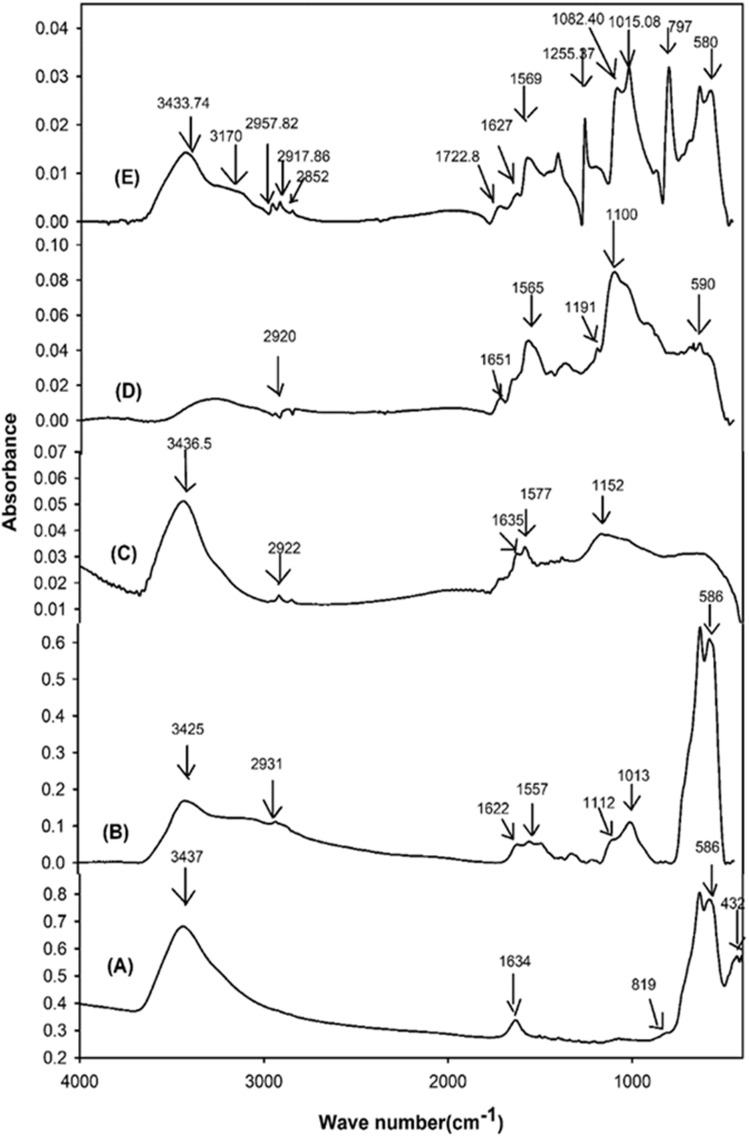
FTIR spectra of (A): Fe_2_O_3_, (B): APTS functionalized Fe_2_O_3_, (C): –COOH MWCNT, (D): M-CNT and (E): MPCNT.

FTIR spectra of Fe_3_O_4_, APTS functionalized Fe_3_O_4_, MWCNT-COOH, magnetic MWCNT, and MPCNT were recorded to determine the successful modification of the functional groups present in each component of the magnetic sorbents and are given in Fig. 5. In Fe_3_O_4_ particles, the absorption bands at 432 cm^−1^ and 586 cm^−1^ can be assigned to Fe–O vibration mode. The disappearance of the Fe–O peak at 432 cm^−1^ in Fig. 5B, after coating the iron with APTS, is likely due to the coordination of APTS with the iron surface. The amine groups of APTS may interact with the iron, modifying the Fe–O bond and either shifting or suppressing its corresponding vibrational mode. Additionally, the surface coverage by APTS and the introduction of new functional groups (such as –NH_2_ and Si–O) may lead to new absorption peaks, which could mask or obscure the Fe–O vibration. The peak at 819 cm^−1^ indicates Fe–OH vibration and that at 3425 cm^−1^ is due to the presence of Fe–OH/–OH on the surface of the Fe_3_O_4_ particles.^52^ After functionalization with 3-amino propyl tri ethoxy silane, the –OH peak at 3427 cm^−1^ has been reduced considerably and that at 819 cm^−1^ disappeared completely. Si–O–Si stretching bands can be seen from 1112 cm^−1^ to 1013 cm^−1^. The Si–OH stretching of surface silanols appeared from 3425 cm^−1^ and as the broad peak in the 3700–3000 cm^−1^.–CH_2_- alkyl groups from the silane is seen at 2931 cm^−1^. Two bands at 1622 cm^−1^ and 1557 cm^−1^ are corresponded to the bending vibrations of –NH_2_ and –CH_2_ groups, respectively. All these bands revealed that the surface of the iron-nano particle is modified with APTS^48,53^ Relative standard deviation.

**Fig. 6:**
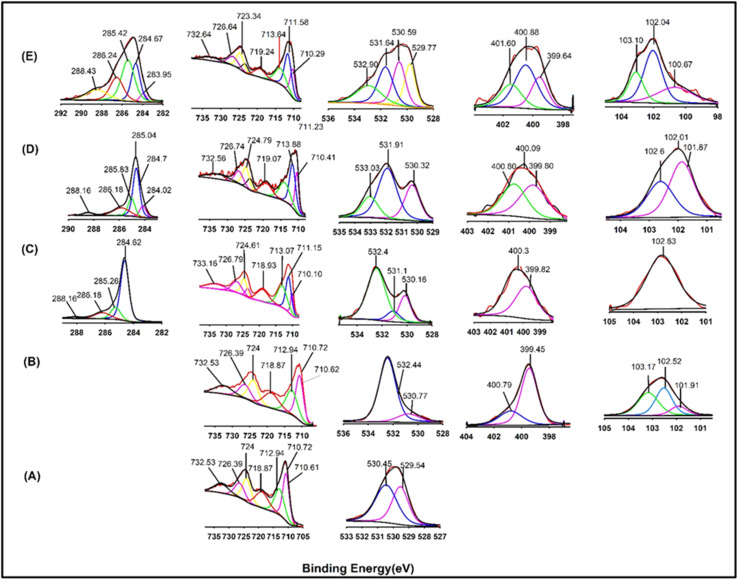
XPS spectra of (A): Fe_3_O_4_, (B): APTS functionalized Fe_3_O_4_, (C): magnetic-CNT, (D): magnetic-PCNT and (E): magnetic-PTCl.

XPS analysis is very efficient for surface analysis of materials, and it is done for Fe_3_O_4_, APTS functionalized Fe_3_O_4_, M-CNT, MPCNT, and MPTCl to make sure about the successful particle synthesis as it is depicted in the scheme. The XPS peaks are given in Fig. 6. Deconvolution was carried out to find out the functional group present in each atom. The XPS peaks of Fe 2p_3/2_ and Fe 2p_1/2_ for the Fe_3_O_4_ sample are shown in Fig. 6A. The peak values of Fe 2p_3/2_ has been studied earlier by several researchers and have made a conclusion that the peak values comes in between 710.5 eV and 711.2 eV and the two peaks of Fe 2p_3/2_ is narrower and stronger than Fe 2p_1/2_ and the area of Fe 2p_3/2_ peak is greater than that of Fe 2p_1/2_ which is situated at 724 eV.^54–58^ The Fe 2p_3/2_ peak has associated satellite peaks whose binding energy is more than that of the Fe 2p_3/2_ peak.^56^ In the given Fig. 6, satellite peaks at 718.87 eV (Fe_3_O_4_) shifted to higher binding energy 719.07 eV (MPCNT) and 719.24 eV (MPTCl) which might be due to the interfacial interaction resulted due to the sorbent formation. Carbon presence in negligible amount was there in both Fe_3_O_4_ and in APTS functionalized Fe_3_O_4_. The peak 529.54 eV is assigned to O–Fe bonding and that at 530.45 eV indicates O–H bonding. The O–H bond was diminished after APTS functionalization as like it was expected. The reduction of O–H peaks and the presence of nitrogen and silica in the APTS- Fe_3_O_4_ points out more information about successful functionalization. N–C bond is present at 399.45e V and the peak at 400.79 eV is present due to the non-reacted terminal –NH_2_ end group which clearly explains the reaction through the –OR group of APTS. In magnetic MWCNT, the peaks at 284.62 eV, 285.26 eV, 286.18 eV, 288.16 eV are attributed to C–C, C–H, C–O, and C=O respectively. When reacted with acid-functionalized MWCNT, the peak presented at 400.79 eV for the amine terminal group disappeared and a new peak at 400.3 eV for N–H bonding of the amide group between magnetic MWCNT and MWCNT-COOH appeared.^36^ The peak at 530.16 eV in magnetic MWCNT indicated the presence of unreacted OH from the MWCNT-COOH and which was supposed to further react with the terminal amine group of PDMS to form amide linkage in MPCNT. The new peak at 531.10 eV represented the O=C (NHCO) bond, and the O–Si peak which was already there at 532.4 eV after the APTS functionalization of iron still present in magnetic MWCNT provided solid evidence of the reaction between MWCNT-COOH and the APTS functionalized Fe_3_O_4._

The XPS spectra of the MPCNT shows peaks for C, Fe, O, N, and Si as was expected. The MPCNT particle showed new carbon peaks at lower binding energy due to C–H from PDMS and the area of the C=O (NHCO) peak at 286.16 eV was increased due to the reaction with PDMS. A considerable increment in the peak area at 531.10 eV also supported the amide bonding between PDMS and magnetic MWCNT. The unreacted terminal amine is seen at 400.80 eV and N–C and N–H peaks can be seen at 399.80 eV and 400.09 eV respectively.^59,60^ The N 1s spectrum showed a prominent peak at approximately 399.5 eV, which can be attributed to the nitrogen in the amine group (–NH_2_) from the APTS molecule, indicating successful amidation on the iron surface. The appearance of this peak supports the formation of an amide bond between the amine group of APTS and the Fe_3_O_4_ surface. Additionally, shifts in the O 1s peak indicated the incorporation of the silane group, suggesting an enhanced interaction between the APTS and the iron oxide surface. The Fe 2p spectrum showed no significant shift, indicating that the iron oxide core remained intact and did not undergo substantial changes during the functionalization process. These XPS findings confirm the successful functionalization of Fe_3_O_4_ with APTS, which is expected to improve the surface reactivity and provide a suitable platform for further modifications. The peaks at 102.6 eV confirm the Si–O–Si peak and that at 102.01 eV indicates Si–O–C bonding.^61^ The peak at 101.87 represents the Si–O–C peak formed from –COOH CNT. The concomitant increment in the surface area of silicon peaks with respect to the PDMS addition gives more valuable confirmation about the formed polymeric sorbent MPCNT.^60^ The reduction in the peak area at 284.67 eV in MPTCl revealed the absence of MWCNT in that particle. Even more importantly, the considerable increment in the peak area of C=O at 288 eV due to the inclusion of the C=O group of the terephthaloyl chloride again verified the absence of MWCNT. These spectral modifications imply a shift in the Fe d-band center, reflecting changes in the electron density at the surface. Since a downshift in the d-band center is generally correlated with enhanced adsorption of aromatic and phenolic molecules, these results provide a plausible explanation for the higher BPA removal efficiency of MPCNT compared to MPTCl. The elemental composition obtained from XPS is summarized in Table 1S. Both sorbents contained Fe, O, C, N, and Si, consistent with the presence of Fe_3_O_4_, polymer coating, and APTS functionalization. Compared with MPTCl, MPCNT exhibited a marked increase in C and O content, which can be attributed to the incorporation of MWCNT-COOH. The higher nitrogen level in MPCNT also supports successful interfacial bonding through amide and silane linkages. These compositional changes confirm the structural modification of MPCNT and are consistent with its superior sorption performance toward BPA.

**Fig. 7:**
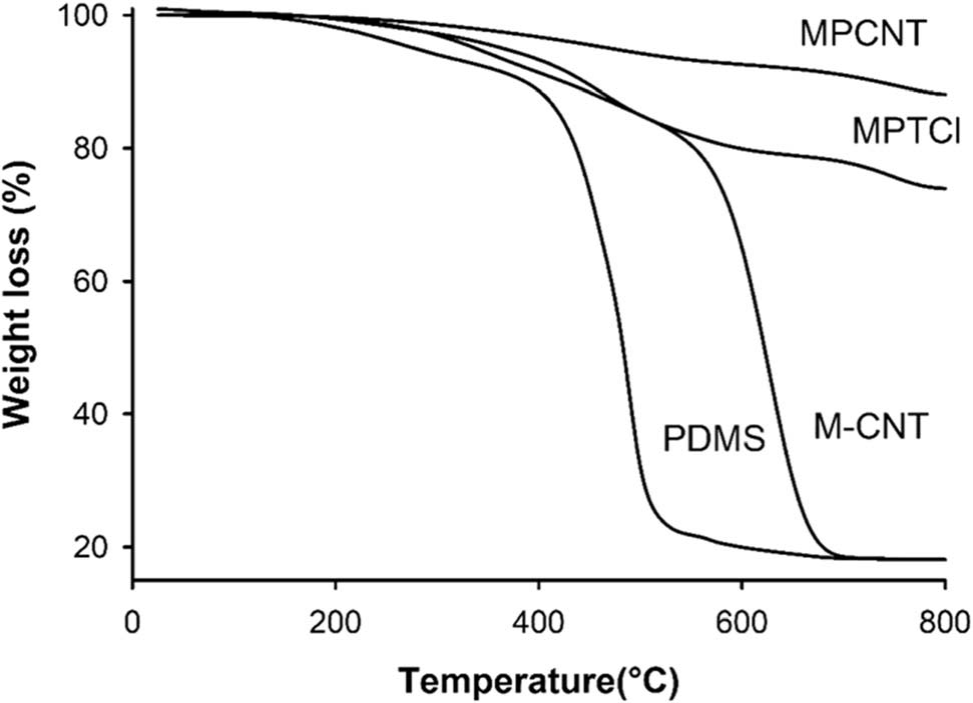
TGA thermograms of PDMS, magnetic-CNT, magnetic-PCNT and magnetic-PTCl.

Thermogravimetric analysis (TGA) measures the changes in weight of a sample with increasing temperature. The weight loss study under air for pure PDMS, MPCNT, and MPTCl were performed over a temperature range of 0–800 °C with a constant heating rate of 10 °C min^−1^. It has been observed in Fig. 7 that thermal oxidative degradation of PDMS took place in two stages, the first stage at 300 °C and the second stage at 497 °C produces a mixture of oligomers. Oligomers again decomposed and the residue was left as silica at 500 °C in the case of uncured PDMS.^62–64^ In the case of magnetic M-CNT, the APTS functionalized Fe_3_O_4_ had a slight decomposition around 400–600 °C due to the decomposition of the magnetic content and the MWCNT decomposition took place at 647 °C.^65^ In MPTCl, the PDMS is cured by terephthaloyl chloride and it caused a considerable increment in the decomposition temperature of the PDMS hybrid.^66^ The APTS functionalized iron particles also could hinder the motion of the volatiles produced by decomposition, to increase the thermal stability of the MPTCl hybrid. It is seen that the initial decomposition values shifted to 457 °C which corresponds to the decomposition of the magnetic content. The 74% residue left at 800 C is due to thermally stable iron moieties and the silica from both PDMS and APTS functionalized Iron oxide.^66^ In MPCNT, the MWCNT decomposes at a very high temperature which provided very high thermal stability of the MPCNT sorbent.^67^ The higher quantity of the residue in the MPCNT could be attributed to the presence of inorganic components of iron in the samples which sustain even at high temperatures, and the silica content along with the undecomposed polymer. The PDMS-wrapped MWCNT may not decompose completely at 800 °C and that too left as residue at 800 °C. Thus, the incorporation of MWCNTs in the polymer matrix resulted in increased thermal stability and residue gaining. All this evidence strongly supported the formation of PDMS functionalized magnetic sorbents.

The effect of pH is crucial for the adsorption studies which determines how the adsorption process takes place and whether the ionic or molecular form of the analyte is getting adsorbed onto sorbent particles. To analyze the surface charge distribution of the synthesized particles, 15 mg sorbent was dispersed in 15 mL of water and sonicated for 10 min. Afterwards, the sample was transferred to the multipurpose titration unit (MPT) for pH titration. The 0.5 M (optimized method) concentration of the acid and base was used for the analysis. The pH range was fixed between 3-12 with an increment rate of 0.5. When the external solution has a greater pH value than that of the hybrid sorbent, it will release protons and become negatively charged. The pI values of M-CNT and that of the sorbent MPCNT are 3.27 and 5.39 respectively as it is seen in Fig. 1S. The difference between the pI values of M-CNT and that of MPCNT revealed the successful modification that happened with the targeted sorbent particle by the polymer addition.^46^

### 3.2. Optimization of the proposed MSPE strategy

The extraction conditions influencing the extraction performance were examined thoroughly to achieve optimum efficiency. A series of experiments were carried out by changing one variable at the same time keeping all other variables at a constant level and the extraction response was monitored by analyzing the average peak area of the corresponding HPLC chromatograms. The optimized parameters include the polymer content, sample volume, sample pH, desorption sample volume, and salting-out effect, and the peak area obtained from the HPLC analysis was used to find out the extraction capacity of the prepared sorbents. The PDMS amount that could give the maximum extraction performance of BPA, for the synthesis of the MPCNT sorbent hybrid was investigated by using different amounts of PDMS and in each case, the average peak area obtained was evaluated. The procedure of the synthesis was carried out as it was described (Section 2.6) previously. To carry out the extraction, 40 mg of hybrid sorbent was used to extract the bisphenol A from a 4 mL aqueous standard solution of 5 mg L^−1^. The mixture was stirred for 30 min in a vortex, and the bisphenol A adsorbed sorbent was isolated by a magnet. The sample liquid was removed and 2 mL of desorption sample of acetonitrile – acetic acid buffer in the ratio 70 : 30 (v/v) was added and vortexed another 30 min. The sorbent separation was done by an external magnet and a 10 µL aliquot was analyzed by HPLC-UV. The ratio of –COOH from MWCNT and the –NH_2_ from PDMS was first taken in stoichiometric ratio to do the sorbent (MPCNT) preparation, and extraction was done with the prepared sorbent. Then the amount of polymer increased in each subsequent preparation keeping the weight of magnetic content and MWCNT-COOH at a constant level and repeated the bisphenol A extraction at pH-9 by using the above-explained procedure. The hybrid sorbent (MPCNT) containing 3.2 mg (30 times to stoichiometric ratio) of PDMS could provide the best results in terms of extraction as it is seen in Fig. 2S. And therefore 3.2 mg was selected as the optimum for the sorbent preparation. From the results obtained, it can be concluded that the polymer amount positively affects the extraction capacity of the MPCNT hybrid. But the polymer in higher amounts after the optimum weight resulted in the reduction of the porosity by imbibing through the pores. This might have caused a considerable reduction in the surface area by increasing the particle size with a reduced number of adsorption sites.

The superior extraction efficiency of MPCNT over MPTCl (Fig. 9) is directly linked to its nanostructure. Quantitative TEM analysis confirmed uniform MWCNT dispersion (85% isolated tubes), maximizing accessible surface area and preventing agglomeration-induced pore blockage. In contrast, agglomerated CNTs would reduce active sites and increase diffusion resistance, lowering BPA uptake. This structural advantage synergizes with the π–π interactions and hydrogen bonding mechanisms discussed earlier.

#### 3.2.1 Effect of pH

A series of extraction and analysis were carried out by changing sample pH (pH-3, pH-7, and pH-9) at the same time keeping all other variables at a constant level and the results were analyzed; pH −9 was producing the best result. The pH dependence of BPA adsorption onto MPCNT is governed by the interplay between BPA speciation and the sorbent's surface charge. BPA (p*K*_a_ = 9.6–10.2) exists predominantly in its neutral molecular form below pH 9.6, while deprotonation occurs above this pH, forming the anionic phenolate species. Zeta potential measurements (Fig. 1S) revealed that MPCNT has an isoelectric point (pI) of 5.39, acquiring a negative surface charge above pH 5.39 due to deprotonation of carboxyl groups (–COOH → –COO^−^) on MWCNT-COOH and silanol groups (–SiOH → –SiO^−^) on PDMS.^68–71^ At pH 9, MPCNT is negatively charged (pI = 5.6), while BPA remains largely neutral. This favors adsorption *via* hydrophobic interactions and hydrogen bonding between BPA's phenolic –OH groups and MPCNT's oxygen-rich functionalities. In contrast, at pH > 10, electrostatic repulsion between anionic BPA and the negatively charged MPCNT surface reduces adsorption efficiency. At acidic pH (*e.g.*, pH 3), protonation of MPCNT's surface groups (*e.g.*, –NH_2_ → –NH_3_^+^) occurs, but BPA's low solubility and weak hydrophobic interactions limit adsorption. Thus, pH 9 optimizes adsorption by balancing BPA's neutrality and MPCNT's moderate negative charge, enabling synergistic non-covalent interactions.

The sorption mechanism of BPA onto MPCNT is governed by multiple synergistic interactions arising from the surface modification. The aromatic structure of MWCNT enables π–π stacking with the phenolic rings of BPA, while the –COOH groups on MWCNT establish hydrogen bonding with the hydroxyl groups of BPA. Functionalization with APTS introduces amino groups, which can participate in electrostatic interactions depending on the pH of the medium. PDMS contributes hydrophobic domains that interact with the nonpolar backbone of BPA, further enhancing affinity. Morphological analyses (SEM, TEM, AFM) confirmed that MPCNT provided higher surface roughness and porosity compared to MPTCl, increasing the availability of active sites. The pH-dependent extraction behavior and the observed salting-out effect further support the roles of hydrogen bonding, electrostatics, and hydrophobic forces in the adsorption mechanism. Together, these features explain the enhanced extraction capacity of MPCNT relative to MPTCl.

#### 3.2.2 Effect of sample volume

**Fig. 8:**
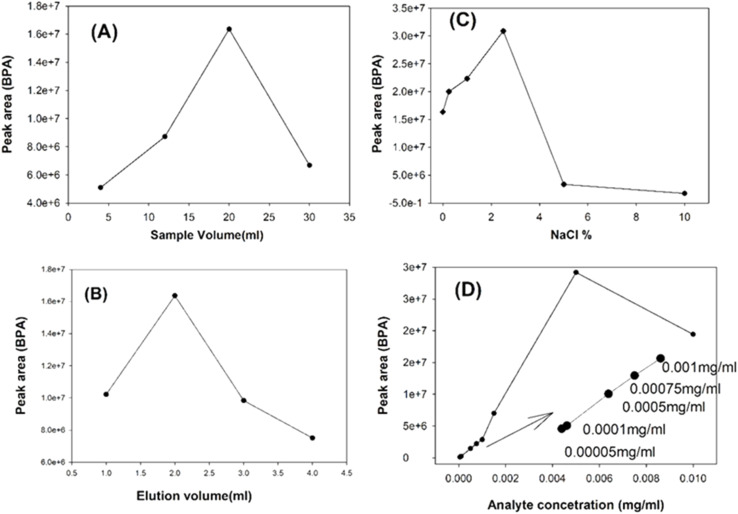
Extraction performance of the magnetic-PCNT sorbent with different variables (A) sample volume, (B) desorption volume, (C) % ionic salt, and (D) initial concentration.

One of the most important factors to be examined for the extraction efficiency of the sorbent materials for the targeted analyte in sample volume. Generally, the extraction efficiency and the pre-concentration factors will rise up with increased sample volume.^70^ When the sample volume increases, the amount of target analyte that is adsorbed on the sorbent also increases and it can enhance the sensitivity of the extraction method. But the time required to reach the equilibrium in the extraction stage was also found to be increased and it was becoming complicated.^69^ Low sample volumes are effective regarding the extraction times and solvent matrix effects.^72^ It is observed that when sample volume increased from 20 mL by keeping the time constant (30 min), the analyte peak area started to decrease. In the present work, it is noticed that the analyte peak area increased from volumes of 4 mL up to 20 mL and after 20 mL it started to reduce the analyte release may due to the time limit. Best recoveries for solid phase extraction sorbents were determined at a loading volume of 20 mL, denoting that the analyte was fully bound towards the active sites. The analyte peak area turned down beyond 20 mL loading volume, since the active adsorption sites on MPCNT were saturated or overfilled, and thus the excessive BPA analyte could not be bound within the sorbent sites before the selected time of 30 min. For volume greater than 20 mL it may need more time as well. As a result, a sample volume of 20 mL was chosen for all the subsequent experiments. The obtained results are depicted in Fig. 8A. The PDMS surface contains not only adsorption sites but also silica and CNTs which are rigid particles, and they will not move or bend. Hence the imbibed polymer chains must get enough time with proper conditions to get adjusted to include more analyte.

#### 3.2.3 Effect of desorption sample volume

The extraction efficiency can be affected by both the elution solvent type and its volume. The analyte peak area can be maximum only when the extraction strategy achieves maximum efficiency. The desorption solvent must be capable to displace the trapped BPA analyte from the MPCNT sorbent at the possible minimum volume and which is why the selection of elution solvent with an appropriate ratio is highly essential.^73^ In this study, 20 mL aqueous BPA solution presented the highest BPA extraction and the addition of 2 mL of 30% acetic acid buffer/acetonitrile (v/v) gave the maximum peak area. Both adsorption and desorption timings were kept constant (30 min.) throughout the study. Acetonitrile was chosen as an elution medium because it was a good solvent of BPA, and the acetic acid buffer was used since the analyte pH after the adsorption process is high due to the used adsorption solvent, at pH 9. Hence the acetic acid buffer-acetonitrile mixture was selected which could induce efficient elution. The adsorption volume at pH 9 helped to surmount the strong interactions between MPCNT sorbent and the BPA analyte. Acetic acid buffer/ethanol also was used to test the analyte peak area but the acetic acid buffer-acetonitrile mixture was providing the best BPA peak area with different ratios of acetic acid. When a 10% acetic acid buffer was used, the yield was too low. To assess the effectiveness of the acetic acid buffer in facilitating BPA adsorption during the desorption process, a 0% buffer solution was also evaluated. Therefore, acetonitrile with 30% acetic acid buffer was chosen as the best solvent for elution. The next step was to optimize the elution solvent volume and to fulfill that purpose different volumes (1 mL, 2 mL, 3 mL, and 4 mL) of elution solvents were evaluated. Using 1 mL of eluent gave a lower peak area, as it was insufficient to fully desorb BPA from the sorbent surface. With 2 mL eluent, the BPA peak area reached its maximum and was again found to be decreased with 3 mL and 4 mL. Results indicated that BPA peak area was significantly increased by 2 mL eluent volume with 30% acetic acid buffer in acetonitrile as it is seen in Fig. 8B.

#### 3.2.4 Salt-tolerant ability

Salting-out in aqueous NaCl solutions is relevant for the extraction behavior of organic analytes. The addition of a salt to the aqueous solution can decrease the solvent (water) amount to dissolve the analyte. In another word, the solubility of the analyte decreases due to salt addition, and it will accelerate the analyte release to the sorbent surface and thus increases the efficiency of the extraction method. This happens because of the formation of hydration spheres over ions that formed from the salt molecules.^70,74–76^ The hydrogen bonds also play a prominent role in the binding of the BPA analyte to MPCNT, and it was necessary to find out the optimum salt amount to support the extraction process.

In the present work, various amounts of NaCl in the aqueous phase in different ranges (0, 0.25, 1, 2. 5, 5, and 10 (w/v)) were tested to study the effect of NaCl addition on extraction performance. As the Fig. 8 C illustrated, the maximum analyte was released to MPCNT by the addition of 2.5% NaCl. It is noticed that the chromatographic peak area was decreased beyond 2.5% and it was selected as the optimum dosage.

#### 3.2.5 Effect of analyte concentration

Not only the amount of the solvent but also the concentration of the loading solvent is also a matter that must be considered since the more selective steps of the MSPE procedure will provide more sensitive results. The extraction performance should not depend on the concentration of the sample. In other words, there ought to be no prominent difference in the BPA analyte extraction at all ranges of concentrations that are analyzed. In this MSPE study, above all optimizations were done for 0.05 mg L^−1^ concentration of BPA and it was noted that the peak was too wide in shape after the addition of salt. Therefore, the effect of BPA concentration on the extraction efficiency of the MPCNT sorbent was evaluated by using the concentration range 0.05 to 1 mg L^−1^ whereas other experimental conditions were kept constant. Without salt addition, the peak area for 0.05 mg L^−1^ concentration was increased by 88.8% with 2.5% (NaCl to aqueous phase, w/v) and the peak became flat. The concentration 0.05 mg L^−1^ also gave large and wide chromatogram peaks of BPA. The BPA peaks for the lower sample concentrations were found to be good and sharp. The sample concentration 1 mg L^−1^ was chosen after the optimization study to carry out the BPA extraction with MPTCl. The selected conditions are given in Table 2S.

**Fig. 9:**
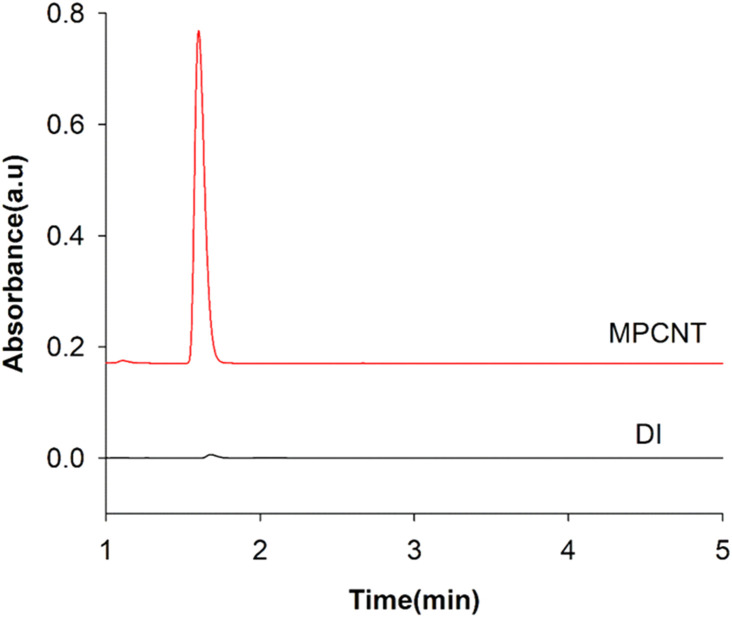
HPLC chromatogram of 1 mg L^−1^ of BPA direct injection and after extracted by magnetic-PCNT.

The peak area obtained from the HPLC analysis after the BPA extraction by MPTCl sorbent was less than that obtained by MPCNT. The reduction in the peak area may be due to the lesser adsorption site areas in the MPTCl sorbent as was explained in SEM analysis. The presence of MWCNT might have supplied more adsorption sites by providing voids in the PDMS matrix and this could lead to more extraction comparatively (Fig. 9).

The improved extraction performance of MPCNT can be attributed to the multiple interaction mechanisms introduced by the incorporation of MWCNT-COOH. First, the π–π interactions between the aromatic rings of BPA and the conjugated graphitic surface of MWCNTs facilitate strong adsorption. Second, the –COOH functional groups present on MWCNTs enable hydrogen bonding with the hydroxyl groups of BPA, further enhancing affinity. Third, SEM, TEM, and AFM analyses confirmed that the inclusion of MWCNTs increased particle roughness and created additional porous channels, thereby expanding the available surface area and adsorption site density. The observed pH dependence of adsorption supports the role of hydrogen bonding and electrostatic effects, while the improvement in recovery with moderate salt addition is consistent with hydrophobic and π–π interactions. Although adsorption isotherm and kinetic modeling were not conducted in this study, the combined structural and optimization data strongly indicate that the synergistic effects of π–π stacking, hydrogen bonding, and increased surface area are responsible for the enhanced BPA uptake by MPCNT compared to MPTCl. Future work will focus on applying adsorption isotherm and kinetic models to quantify these interactions and provide deeper mechanistic insights.

### 3.3. Reusability of the synthesized sorbents

To study the reusable properties of the synthesized sorbents MPTCl and MPCNT, the extraction process consisting of the selected adsorption/desorption cycles was conducted. After each extraction cycle, the sorbents were washed three times thoroughly with absolute ethanol and 2 times with acetonitrile to remove the trapped bisphenol A and other impurities. Fig. 3S indicates regeneration yields of the developed sorbents during the five cycles. As visibly noticed from the results, extraction yields remained steady up to the first five cycles in the case of MPCNT. This indicates that the prepared MPCNT has excellent repeatability or recycling performance for the BPA removal from aqueous solutions as it seemed relatively stable throughout the experiments. Even more importantly it reveals the significance of carbon nanotubes as the backbone supporter of MPCNT for the reusable behavior.

Reusability is an essential property of an ideal extractant. Under optimal conditions, MPTCl was also applied to the sequential extraction procedure, the BPA-loaded extractant was magnetic separated, eluted, washed, dried, and recycled, and repeatedly. The recovery percentage was found to be [95% ± 0.1] after the first cycle and remained consistent across five cycles, indicating minimal loss during the recovery process. From the graphic illustration in Fig. 3S, after the first recycling, the extraction efficiency of MPTCl decreased considerably. It might be attributed to the structural difference that the MPTCl possesses due to the CNT absence.^35,77^

The operational stability of MPCNT can be inferred from its structural characterization and reusability performance. Thermogravimetric analysis (TGA, Fig. 7) revealed MPCNT maintains structural integrity up to 300 °C, with 74% residue at 800 °C, indicating excellent thermal stability far beyond typical extraction temperatures (25–80 °C). FTIR spectra (Fig. 5D) showed no degradation of characteristic amide bonds (C=O at 1650 cm^−1^) or Si–O–Si linkages (1013–1112 cm^−1^) after synthesis, suggesting chemical robustness. XPS data (Fig. 6D) further confirmed stable covalent bonding between MWCNT-COOH and PDMS, with no significant elemental composition changes observed.

The presence of MWCNT-COOH critically enhances stability compared to MPTCl. Covalent amide bonds between MWCNT-COOH and PDMS (evidenced by FTIR/XPS) provide superior resistance to hydrolysis and mechanical stress *versus* the ester linkages in MPTCl. This is reflected in MPCNT's consistent extraction efficiency over 5 cycles (Fig. 3S), while MPTCl showed earlier performance decline. Zeta potential measurements (Fig. 1S) confirmed MPCNT maintains surface charge stability across pH 3–12, supporting its chemical resilience. Collectively, these data indicate MPCNT operates stably under typical extraction conditions (25–80 °C, pH 3–12), with MWCNT-COOH playing a key role in preventing degradation.

### 3.4. Analytical performance

Relative standard deviation (%RSD) of the proposed method were examined by five repeated extractions with BPA (0.05 to 1 mg L^−1^). The relative standard deviation (% RSD) was calculated by dividing the standard deviation by the mean and multiplying by 100.^78^ The results revealed acceptable reproducibility with % RSD = 7.85% (*n* = 5). The method showed a linear response from 0.05 to 1 mg L^−1^ (*r*^2^ = 0.9992). The limit of detection (LOD) and limit of quantification (LOQ) were estimated at 15.15 µg L^−1^ and 50.00 µg L^−1^, respectively, based on the lowest concentration validated within the linear range. The calibration graph obtained by using the MPCNT for BPA analyte was linear (*r*^*2*^ = 0.9992). As compared to recently published research (Table 1), the results that have been achieved by the magnetic-PDMS-CNT sorbent are encouraging to be further expanded and investigated for the extraction and enrichment of BPA.

**Table 1:** Comparison of the present work with some SPME previously reported methods

Samples	Sorbent	Reusability (cycles)	Synthesis complexity	LOD	LOQ	Detection	Ref
Water	CQD@CF (carbon quantum dots)	∼15	Moderate (*in situ* synthesis)	0.01 ng mL^−1^	0.04 ng mL^−1^	HPLC – UV	79
Water, food, paper	Molecularly Imprinted pPolymers (MIP)	Up to 6	Moderate (polymerization)	0.015 µg mL^−1^	0.045 µg mL^−1^	HPLC-fluorecence	80
Water, food	β-cyclodextrin/m-β-cyclodextrin complexes & SPE	Not typically reused	Low (complex formation)	0.13–0.38 µg mL^−1^	0.13–0.38 µg mL^−1^	Fluorescence	80
Baby bottles, water	SPME (PA fiber, derivatization)	Single use	Low (commercial fiber)	0.003–0.016 µg mL^−1^	—	GC-MS	81
Water	Magnetic peptide bead	∼6 cycles	Moderate	Not stated	Not stated	Adsorption/HPLC	82
Soft drinks	MWCNT-Fe_3_O_4_ MSPE	Up to 5–6	High (nanomaterial)	0.001 ng mL^−1^	0.0035 ng mL^−1^	GC-MS	83
Water, food, paper	MIP	Up to 6	Moderate	0.015 ng mL^−1^	0.045 ng mL^−1^	HPLC-fluorecence	84
Drinks, water, baby bottles	SPME (PA fiber, derivatization)	Single use	Very low (commercial)	0.003–0.016 ng mL^−1^	—	GC-MS	81
Acetate buffer	Magnetic-PDMS-CNT	≥5	Moderate (4 steps, 24 h)	15.15 µg L^−1^	50 µg L^−1^	HPLC-UV	This work

## 4 Conclusions

From the detailed elucidation based on the thriving formation of the hybrid sorbents and its extraction analysis, it is obvious that the combination of unique structures, dimensions, and surface morphologies make CNT an interesting material to synthesize regenerative hybrid sorbents for extraction purposes. The capacity of a sorbent depends on the amount of surface area to which the analyte can adhere as well as the type of surface. In MPCNT sorbent there were more regenerative adsorption cites which could retain the extraction power up to five cycles. The extraction performance of the sorbents was assessed in terms of LOD and LOQ. The MPCNT sorbent achieved efficient BPA extraction in a competitive acetate buffer matrix, with low LOD (15.15 µg L^−1^), high reproducibility (RSD = 7.85%), and reusability over five cycles. The use of acetate buffer as a competitive medium confirmed the sorbent's resilience against ionic interference. The prepared MPCNT sorbent allowed acceptable levels of the above obtained analytical parameters and this sorbent preparation procedure with suitable polymer is expected to be useful for the analysis and enrichment of other plastic compounds.

## Conflicts of interest

The authors declare no conflict of interest.

## Supplementary Material

RA-015-D5RA04923G-s001

## Data Availability

Raw data that support the findings are available from the corresponding author upon request. All relevant experimental procedures and results are fully described in the manuscript and supplementary information (SI). Supplementary information is available. See DOI: https://doi.org/10.1039/d5ra04923g.
