# Supplementary material for: Efficient magnetic sorbent for extracting bisphenol A from aqueous samples
Source: RSC Adv. 2025 Dec 5;15(56):48134–48. doi: 10.1039/d5ra04923g (PMC12679565; doi:10.1039/d5ra04923g)
Supplement: RA-015-D5RA04923G-s001 [file RA-015-D5RA04923G-s001.pdf]

Supplementary Information (SI) for RSC Advances.
This journal is © The Royal Society of Chemistry 2025

Efficient Magnetic Sorbent for Extracting Bisphenol A from Aqueous Sample

Abdullah Alhendal,[1]* Jessy Shiju, and Zahoor Ahmad

Department of Chemistry, Kuwait University, P. O. Box 5969, Safat, 13060, Kuwait.

E-mail: abdullah.alhendal@ku.edu.kw.

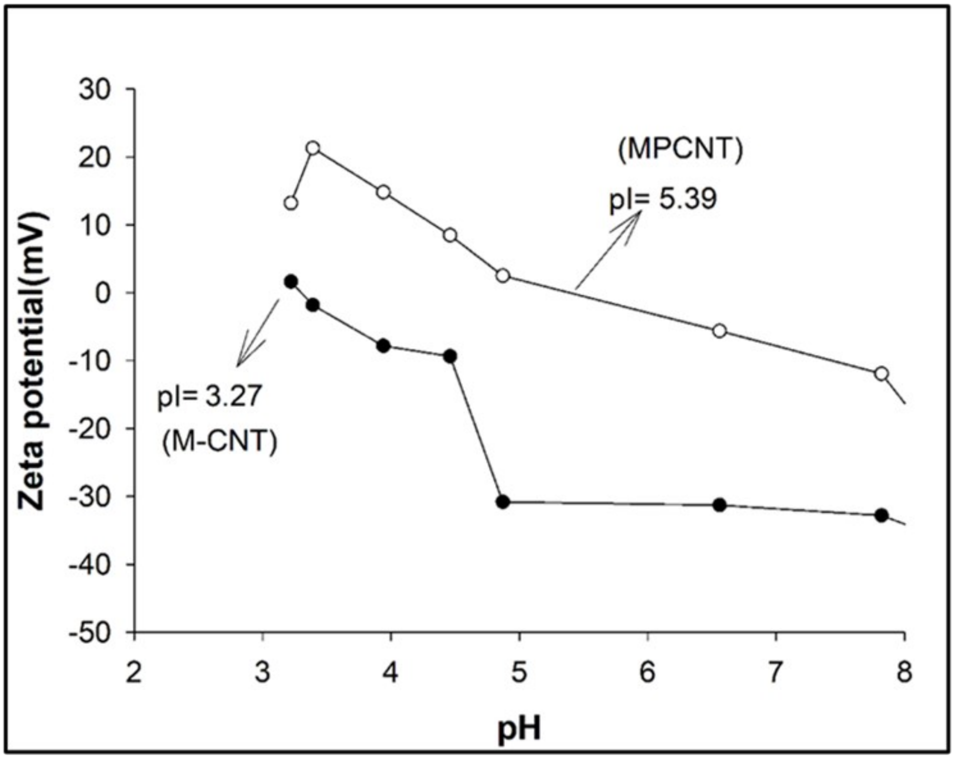

Figure 1S. Zeta potentials of magnetic-CNT and magnetic-PCNT in different pH solution.

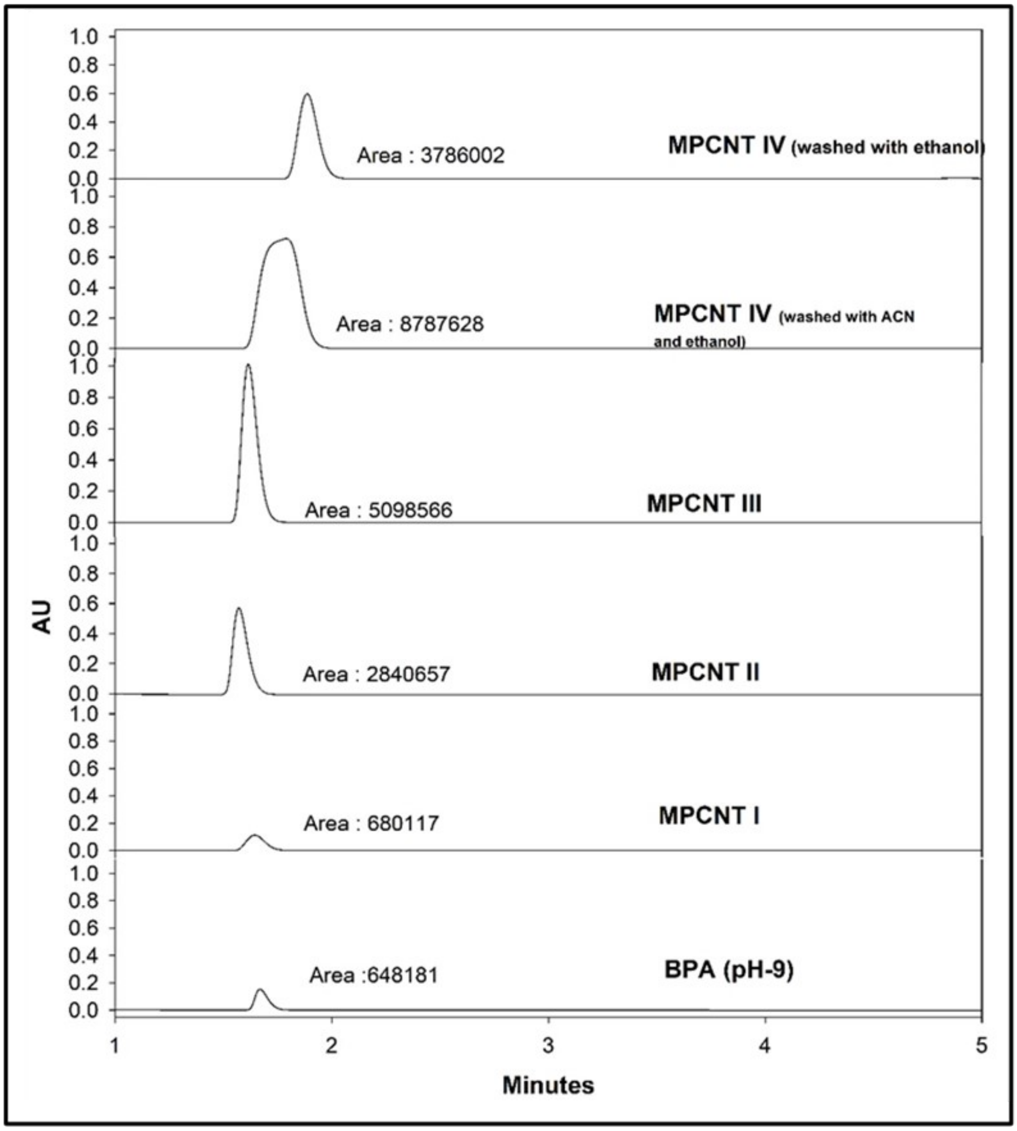

Figure 2S. HPLC chromatograms of MPCNT sorbent with different PDMS amounts.

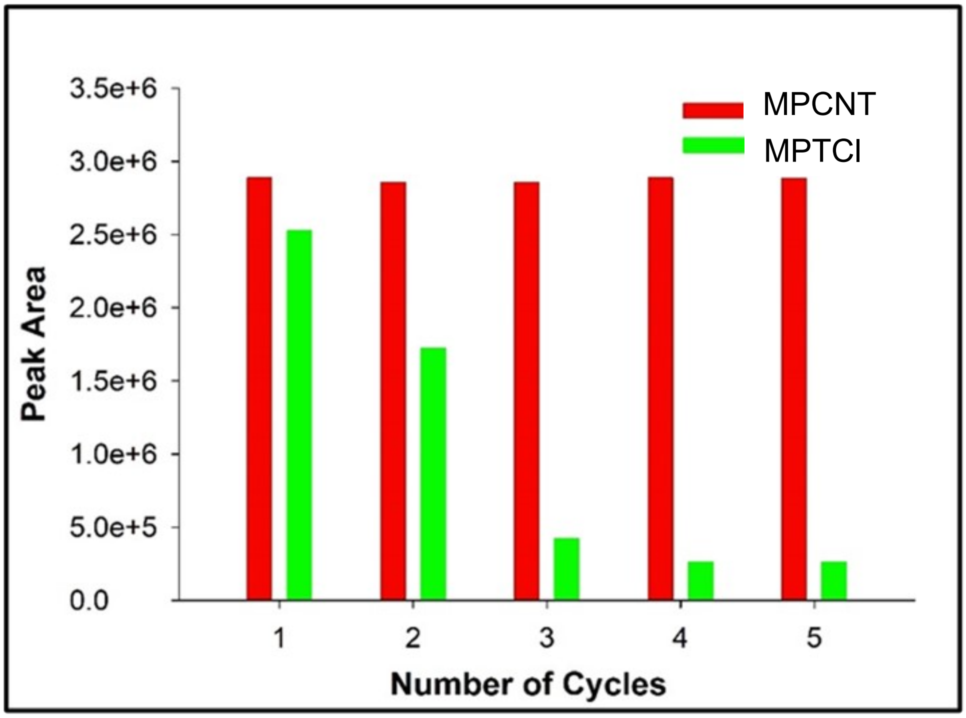

Figure 3S. The recycling performance of magnetic-PCNT and magnetic-PTCl for the bisphenol A extraction by the optimized MSPE procedure.

Table 1S. Selected variables of the MSPE procedure.

**Parameters**	**MPCNT**	**MPTCL**
Sorbent weight	40 mg	40 mg
Adsorption time	30 min	30 min
Desorption time	30 min	30 min
Adsorption solvent details	20 mL aqueous BPA solution of pH-9 with 2.5% NaCl	20 mL aqueous BPA solution of pH-9 with 2.5% NaCl
Elution solvent details	2 mL of 30% acetic acid buffer/acetonitrile (v/v) mixture	2 mL acetic acid buffer/acetonitrile (v/v) mixture
Analyte concentration	1 mg L^{-1}	1 mg L^{-1}
